# Identification of Nutritional Components in Black Sesame Determined by Widely Targeted Metabolomics and Traditional Chinese Medicines

**DOI:** 10.3390/molecules23051180

**Published:** 2018-05-15

**Authors:** Dandan Wang, Liangxiao Zhang, Xiaorong Huang, Xiao Wang, Ruinan Yang, Jin Mao, Xuefang Wang, Xiupin Wang, Qi Zhang, Peiwu Li

**Affiliations:** 1 Oil Crops Research Institute, Chinese Academy of Agricultural Sciences, Wuhan 430062, China; beyondan_good@163.com; 2 Key Laboratory of Biology and Genetic Improvement of Oil Crops, Ministry of Agriculture, Wuhan 430062, China; xiaorongsara@163.com (X.H.); yanrinannan@126.com (R.Y.); 3 Laboratory of Quality and Safety Risk Assessment for Oilseed Products (Wuhan), Ministry of Agriculture, Wuhan 430062, China; 4 Key Laboratory of Detection for Mycotoxins, Ministry of Agriculture, Wuhan 430062, China; zhangqi52x@126.com; 5 Quality Inspection and Test Center for Oilseed Products, Ministry of Agriculture, Wuhan 430062, China; wangxiao0613@163.com (X.W.); maojin106@whu.edu.cn (J.M.); wxf0911@163.com (X.W.); xiupinwang@163.com (X.W.); 6 Hubei Collaborative Innovation Center for Green Transformation of Bio-Resources, Wuhan 430062, China

**Keywords:** sesame, widely targeted metabolomics, differential metabolites, specific nutrients, LC-MS/MS

## Abstract

Chemical composition of secondary metabolites is of great importance for quality control of agricultural products. Black sesame seeds are significantly more expensive than white sesame seeds, because it is thought that black sesame seeds are more beneficial to human health than white sesame seeds. However, the differences in nutrient composition between black sesame seeds and white sesame seeds are still unknown. The current study examined the levels of different metabolites in black and white sesame seeds via the use of a novel metabolomics strategy. Using widely targeted metabolomics data, we obtained the structure and content of 557 metabolites, out of which 217 metabolites were identified, and discovered 30 metabolic pathways activated by the secondary metabolites in both black and white sesame seeds. Our results demonstrated that the main pathways that were differentially activated included: phenylpropanoid biosynthesis, tyrosine metabolism, and riboflavin metabolism. More importantly, the biomarkers that were significantly different between black seeds and white sesame seeds are highly related to the functions recorded in traditional Chinese medicine. The results of this study may serve as a new theoretical reference for breeding experts to promote the genetic improvement of sesame seeds, and therefore the cultivation of higher quality sesame varieties.

## 1. Introduction

Sesame (*Sesamum indicum* L.) belongs to the genus Flax, an important ancient oil crop with a long history of more than 2200 years of cultivation. Sesame is widely distributed in the N40°–S40° tropical and temperate regions [1]. Based on the different colors of the seed coat, sesame seeds are divided into white sesame, black sesame, and yellow sesame, of which white sesame and black sesame seeds are the most common. The breeding of new sesame varieties is important to the growth of sesame industry, and the development of high-yield, high-quality, multi-resistant varieties is a goal of sesame breeding experts. Compared with traditional breeding, molecular breeding incorporates a combination of modern biotechnological methods with classical genetic breeding methods, and include phenotypic screening and genotyping, orienting improved plant genetic traits, and aggregating functional genes to ultimately cultivate new varieties based on the theories of genetics and modern molecular biology. Molecular breeding improves not only breeding efficiency, but can greatly shorten the breeding period [2,3,4], and shows great potential for improving production and quality, and enhancing resistance. Genome breeding is a product of molecular breeding in the era of high throughput sequencing [5,6,7].

With the increase in health awareness, people are more concerned with nutrition and the quality of sesame products. Improvement of yield and increased resistance of sesame seeds to external stressors are not sufficient strategies to meet the current needs of the market. At present, agricultural scientists are changing focus from quantity to quality and efficiency. In recent years, breeding experts have begun to explore the improvement in the quality of crops such as maize [8], soybean [9,10], tomato [11,12], and rapeseed [13] through a variety of breeding methods. However, there are few reports focused on the quality of sesame breeding. The major sesame-producing countries take note of research focused on quality improvement of sesame and its utilization. In the process of sesame breeding, quality breeding is much more valuable than yield breeding. To be consistent with the high protein and low-fat dietary trend, sesame seeds might be produced that contain high protein, low oil, and high melanin [14]. Quality breeding is not only the specific application of black sesame genome breeding, but it is a prerequisite for the cultivation of high-quality sesame varieties via the uncovering of specific functional metabolic processes of black sesame seeds. The cultivation of new varieties of high-quality sesame is of great significance to improving health and nutrition, promoting market consumption, stimulating economic efficiency, and accelerating the growth of the sesame industry. Consequently, quality breeding is a focal point for sesame research world-wide.

Metabolomics explores the dynamic changes of metabolites, the accumulation patterns and genetic origins of plant metabolites [15], and the identification and pathway analysis of metabolically related genes, based on the characteristics of high-throughput, multiple variable, dynamic experiments and statistical analysis [16,17,18,19,20,21,22]. Among them, widely targeted metabolomic analysis is a novel approach that combines the advantages of non-targeted metabolomics and targeted metabolomics. It can simultaneously quantify hundreds of known metabolites and nearly 1000 known and unknown metabolites by using the innate Q TRAP mass spectrometry in multiple reaction monitoring (MRM) mode [23,24]. Consequently, identification of metabolites and elucidation of relevant metabolic processes may be carried out. In these types of studies, qualitative and quantitative analyses could be performed with the goal of determining the pathways governing metabolism in plants. This would be helpful for further studies of gene function, metabolic network analysis, and the functional analysis of small molecules, which could lead to the improvement of crop quality, enhancement of crop yield and resistance, as well as natural drug development. Using metabolic techniques, the previous study also identified a candidate gene related to the oil content and yield of sesame, and the enzymes involved in fatty acid biosynthetic pathways [25]. Given current technology, it may not be difficult to discover biomarkers in agricultural products using metabolomics. However, it should be noted that it is hard to determine the relationships between these biomarkers and quality. Therefore, there is still a need to clarify the precise molecular mechanisms underlying the medicinal and nutritional functions of black sesame, and to develop an evaluation technique and digital representation of black sesame nutrition.

According to traditional Chinese medicine, the term “high quality” refers to the continuous quality improvement of related products, which can play a role in health care, the treatment of disease, and the promotion of human health based on the long-term clinical experience provided by Chinese medical practitioners [26]. Black sesame is not only nutritious, but can be used as medicine in the treatment of various diseases [27]. Black sesame has been shown to play a role in the inhibition of myocardial remodeling and protection of cardiovascular function [28], and may prevent the occurrence and development of atherosclerosis [29], and reduce total cholesterol (TC), low density lipoprotein (LDL) levels, and blood lipids [30]. Black sesame seeds also have a significant protective effect with respect to chronic liver injury [31], they exhibit antioxidant, anti-inflammatory [32], anti-tumor, anti-cancer [33,34], and anti-aging properties [35], and have been shown to protect against neurodegeneration [36]. Studies also reported that black sesame significantly decreased antioxidant stress, exhibiting protective effects in kidneys [37] and preventing osteoporosis [38,39].

The purpose of this study was to conduct widely targeted metabolic analysis of black and white sesame seeds, and to link molecular breeding of high quality sesame with traditional Chinese medicine by determining the important metabolites of black sesame seeds related to the specific nutritional characteristics known in traditional Chinese medicine. The results of this analysis may provide a theoretical basis for the molecular breeding of high-quality black sesame, and may guide the development and genetic enhancement of high quality sesame varieties.

## 2. Results and Discussion

### 2.1. Widely Targeted Metabolic Profiling of Sesame Seeds Based on LC-MS/MS

Qualitative analysis was performed using the stepwise MIM-EPI (multiple ion monitoring-enhanced product ions) strategy and the MS2T data library [23,40]. Metabolomics data of black and white sesame seeds were processed using System Software Analyst (Version 1.6.1 Applied Biosystems Company, Framingham, MA, USA). Metabolites were quantitatively analyzed following collection of secondary data using the MRM model, and information about the content and structure of 557 metabolites found in black and white sesame seeds was obtained. Based on the metabolome database of Wuhan Maiteville Biotechnology Co., Ltd. (Wuhan, China), and the public mass spectrometry databases of MassBank, KNAPSAcK, HMDB, MoTo DB, and METLIN, 557 metabolites were qualitatively analyzed, and the potential structures of 217 metabolites were putatively determined (Supplementary Table S1).

Analysis of metabolic networks linked to the identified 217 metabolites determined that widely targeted metabolite profiling of black and white sesame seeds, based on LC-MS/MS, encompassed the following: taurine and hypotaurine metabolism; alanine, aspartate and glutamate metabolism; arginine and proline metabolism; isoquinoline alkaloid biosynthesis; phenylalanine metabolism; tyrosine metabolism; tryptophan metabolism; sulfur metabolism; indole alkaloid biosynthesis; cysteine and methionine metabolism; and glycine, serine and threonine metabolism. As shown in Table 1, results included the metabolic pathways of common secondary metabolites found in the Kyoto Encyclopedia of Genes and Genomes (KEGG), which is helpful in determining the comparative advantages of nutritional functions of black sesame seeds.

### 2.2. Multivariate and Cluster Analysis of Black and White Sesame Seeds

**Figure 1:**
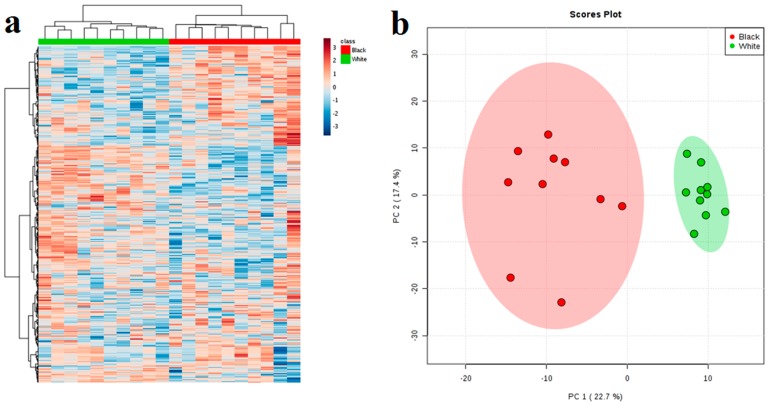
(**a**) Heatmap for black (red block) and white (green block) sesame seeds; (**b**) PCA scores plot for black (red circle) and white (green circle) sesames. Black and white sesame seeds could be completely classified into two classes by using 557 metabolites, indicating that significant differences of metabolites exist between black and white sesame seeds.

Based on the results of metabolomics, 557 compounds isolated from the extracts and 217 extrapolated compounds were analyzed. To eliminate the effect of concentration on pattern recognition, the logarithm (log_10_) of the peak area matrix of black and white sesame metabolite was performed, followed by Poisson normalization [41]. Next, cluster analysis of the metabolite profile of black and white sesame seeds based on LC-MS/MS was performed. According to the results shown in Figure 1, black sesame seeds and white sesame seeds are clearly divided into two categories; values for the black and white sesame seeds were separated in the PCA score plot of sesame metabolites. In addition, they were also clearly divided into two classes on the heatmap, indicating significant differences in the content of the secondary metabolites of black sesame and white sesame seeds.

### 2.3. Identification of Biomarkers of Black Sesame Seeds

Volcano plot was employed to screen the important biomarkers of black sesame seeds. Moreover, the volcano plot graphically displaying two important indicators, *p*-value and fold change, can be used to visually screen differential compounds between two groups of samples. In the current study, two important indicators obtained by the Student’s *t* test (*p*-value and fold change) were used to produce a volcano plot. The negative logarithm of the *p*-value from the Student’s *t* test (-log_10_ (*p*-value)) was the ordinate, and log_2_ (fold change) was the abscissa. If *p*-value is less than 0.01 and the fold change is greater than twice or less than half, significant difference of the metabolite exists between black sesame and white sesame. As results, 69 metabolites were significantly different between black and white sesame seeds. Among them, 20 metabolites were putatively identified and shown in Table 2.

### 2.4. Metabolic Pathway Analysis of Differential Metabolites

**Figure 2:**
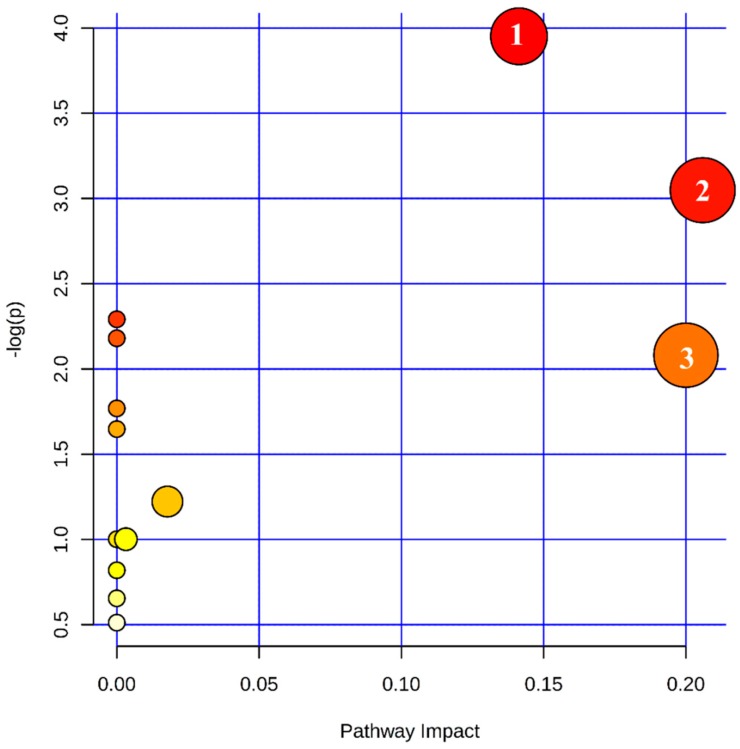
Overview of pathway analysis of white and black sesame. Significantly different metabolites are from three pathways including (1) phenylpropanoid biosynthesis; (2) tryptophan metabolism; (3) riboflavin metabolism.

The KEGG metabolic pathway database is a powerful tool for conducting metabolic analysis and metabolic network research, as it displays a variety of cellular synthesis and degradation processes in the form of diagrams. The final results shown in Figure 2 were obtained using the KEGG metabolic pathway database, metabolite set enrichment analysis (MSEA), or pathway analysis (MetPA) by adopting Global Test algorithm and Pathway Topology Analysis by adopting Relative-between Centrality algorithms.

The vertical axis (−log(P)) indicates the significance of the metabolic pathway enrichment. The deeper the color, the more significant the change in the metabolites in the corresponding pathway. The horizontal axis indicates the impact of the pathway obtained by Pathway Topology Analysis. The larger the circle, the higher the centrality of the metabolite in the corresponding pathways.

The metabolic pathway analysis shown in Figure 2 revealed that the metabolic pathways differentially altered between black sesame and white sesame seeds mainly included phenylpropanoid biosynthesis, tyrosine metabolism, and riboflavin metabolism.

Tyrosinase, a critical enzyme in the synthesis of melanin, determines the rate and yield of melanin production [42]. This enzyme catalyzes the first two steps in the melanin synthesis pathway: L-tyrosine is hydroxylated to form l-DOPA, which is then oxidized to form dopaquinone [43]. Downstream of these reactions, dopachrome may be produced by tyrosinase, which may then be enzymatically converted to 5, 6-dihydroxyindole-2-carboxylic acid by tyrosinase-related protein 2 (TRP-2). Further, 5, 6-dihydroxyindole-2-carboxylic acid may be further oxidized to form indole-5, 6-quinone carboxylic acid by TRP-1 [44]. This result may explain the difference in color between black sesame and white sesame seeds, from the perspective of metabolomics. Wang and Wei et al. [4,45] found the different metabolic pathways and key genes between black sesame and white sesame seeds from the gene level major, including phenylpropane metabolism, tyrosine metabolism causing the differences of sesame seeds color, and the biosynthesis of polyphenol oxidases and flavonoids with the use of genomics knowledge. The differential substances and metabolic pathways that we found from the metabolomics level were consistent with the metabolic pathways from the perspective of genomics.

### 2.5. Nutritional Components Identified in Black Sesame Seeds

**Figure 3:**
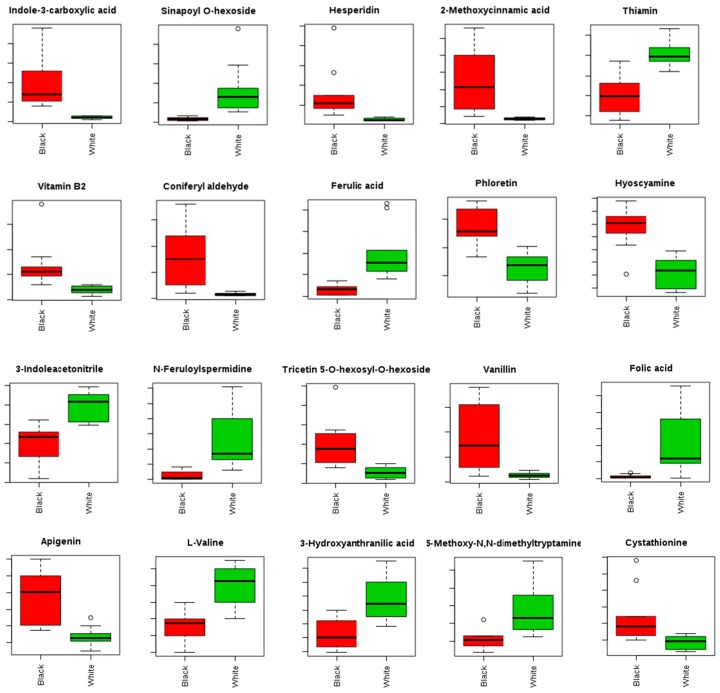
Differences in the contents of 20 metabolites in black (left) and white (right) sesame seeds.

Twenty significantly different compounds in black and white sesame seeds were selected for analysis, and the differences in content are shown in Figure 3. The results showed that the content of indole-3-carboxylic acid, hesperidin, 2-methoxycinnamic acid, vitamin B_2_, coniferyl aldehyde, phloretin, and hyoscyamine were significantly higher in black sesame seeds than in white sesame seeds. According to previous studies, the specific metabolites of black sesame seeds are biologically active. For example, hesperidin has been shown to exhibit protective effects in the nervous and cardiovascular systems, and anti-oxidative, antibacterial, anti-inflammatory, and anti-cancer properties [46,47], while 2-methoxycinnamic acid exhibits antibacterial activity and may inhibit the proliferation and differentiation of cells associated with the regulation of human osteosarcoma MG-63 [48,49]. Meanwhile, vitamin B_2_ is a metabolite that may have protective properties for eyesight, regulate the metabolism of sugar, fat, and protein, and possesses anti-oxidative and anti-infective properties [50,51]. The discovery of these differential metabolites contributes to the functional and nutritional evaluation of black sesame seeds.

## 3. Conclusion

In this study, we studied the comparative nutritional value of black sesame seeds and white sesame seeds by conducting an analysis of widely targeted metabolomics based on LC-MS/MS data. This analysis identified the metabolic pathways of common secondary metabolites in black sesame seeds. The comparison of black sesame and white sesame seeds identified a significant difference in the presence of 20 metabolites. The results of the metabolic pathway analysis indicated that the metabolic pathways that were significantly different between black sesame seeds and white sesame seeds included: phenylpropanoid biosynthesis, tyrosine metabolism, and riboflavin metabolism. This study also determined that the content of indole-3-carboxylic acid, hesperidin, 2-methoxycinnamic acid, vitamin B_2_, coniferyl aldehyde, phloretin, and hyoscyamine were significantly higher in black sesame seeds than in white sesame seeds, which showed a close relationship to characteristic nutritional functions of black sesame seeds recorded in traditional Chinese medicine. This study successfully identified components in black sesame seeds that are consistent with specific characteristics of black sesame seeds reported in other studies and with Chinese traditional medicine experts. The results provide a rationale for the classification of black sesame seeds as a healthy food. Most importantly, this study gives direction for the genomic breeding of sesame and provides important insight for the innovation of high-quality black sesame varieties. 

## 4. Methods

Chromatographic conditions for metabolites were optimized based on the literature [23].

Plant materials: Twenty samples of black sesame seeds and white sesame seeds, which were representative germplasm resources provided by the Sesame Research Group of Chinese Academy of Agricultural Sciences Oil Crops Research Institute, were collected. All sesame seeds were cultivated under the same breeding base and the same growth conditions. The plants were harvested when fully ripe and placed into a freezer at −80 °C. Prior to the experiment, samples of black sesame seeds and white sesame seeds were numbered, crushed with a grinding machine, placed in glass sample vials, and stored in a refrigerator at −80 °C.

Reagents: Methanol, acetonitrile, and acetic acid (HPLC/SPECTRO grade) were purchased in Merck, Germany; Ionized water was obtained by using the Millpore purification system (Millipore, Bedford, Massachusetts, UK); and lidocaine was purchased from BioBioPha company (Kunming, China).

Instruments and supplies: Ultra Performance Liquid Chromatography (Shim-pack UFLC CBM20A) was purchased from SHIMADZU Company (Kyoto, Japan). MS/MS tandem mass spectrometry (4500 Q-TRAP) was purchased from Applied Biosystems Company (Framingham, MA, USA). HSS T3 C18 column (100 mm × 2.1 mm × 1.8 μm) was purchased from Waters Corporation (Milford, MA, USA). Himac CT6E High-Speed Centrifuge was purchased from HITACHI Company, (Tokyo, Japan). BCD-260WDBD type refrigerator was purchased from Qingdao Haier Limited by Share Ltd. (Qingdao, China). Mili-Q ultra-pure water system came from Milipore Company (Burlington, MA, USA). CPA224S electronic analytical balance was purchased from Sartorius Company (Göttingen, Germany). HQ-60 Vortex Mixer was purchased from North Tongzheng Biotechnology Development Company (Beijing, China). Pipettes (10 μL, 200 μL, 1 mL, 5 mL) were purchased from Eppendorf Company (Hamburg, Germany). 0.22 μm organic phase filter was purchased in Millipore Company (Burlington, MA, USA).

### 4.1. Analysis of Sesame Metabolomics Based on LC-MS Data

#### 4.1.1. Sample Preparation

The cryopreserved sesame samples were ground for 1.5 min at 30 Hz using a MixerMill MM 400 (Retsch Technology, Haan, Germany). For extraction, 1.0 mL of 70% methanol containing 0.1 mg/L lidocaine (internal standard) was added to 100 mg of ground sesame seeds and extracted overnight at 4 °C. During this period, the samples were vortexed (10 s, 40 Hz) once every 10 minutes for a total of three times to facilitate the extraction. Following extraction, the pellets were centrifuged at 10,000 g for 10 min. The extracts were filtered through a microporous membrane (0.22 μm pore size) and stored in a sample vial. The quality control sample (QC) was prepared by mixing all of the samples and used to demonstrate the precision of the assay. During the instrumental analysis, a quality control sample was inserted into each of the five test samples to examine the repeatability of the analysis process.

#### 4.1.2. Liquid Chromatographic Mass Spectrometry

The sample extracts were analyzed with the use of an LC-ESI-MS/MS system, which mainly includes HPLC (Shim-pack UFLC SHIMADZU CBM20A system, http://www.shimadzu.com.cn/) and MS (Applied Biosystems 4500 Q TRAP, http://www.appliedbiosystems.com.cn/). API 4500 Q TRAP LC/MS/MS System, equipped with an ESI Turbo Ion-Spray interface, ran in a positive ion mode. Liquid chromatography conditions included the following: (1) The Waters ACQUITY UPLC HSS T3 C18 (100 mm × 2.1 mm × 1.8 μm) chromatographic column was used. (2) Samples were rapidly eluted by using 0.1% formic acid in water (solvent A) and 0.1% formic acid in acetonitrile (solvent B). (3) The separation was achieved with the following gradients: starting with 5% solvent B and raised to 95% B in 11 min, kept 95% B for 1 min, dropped quickly to 5% within 0.1min and kept 5% B for 3 min. (4) Constant flow rate was at 0.4 mL/min, (5) The column temperature was 40 °C, and (6) The injection volume was 5 μL.

The effluents were alternatively connected to an ESI-triple quadrupole-linear ion trap MS/MS (ESI-Q TRAP-MS/MS). LIT (linear ion trap) and triple quadrupole (QQQ) scans were carried out by triple quadrupole-linear ion-trap mass spectrometer (Q TRAP). Mass spectrometry conditions: Electrospray ionization (ESI) temperature was set at 550 °C, mass spectrometry voltage was 5500 V, and gas I (GSI) and gas II (GSII) were set at 55 psi and 60 psi, respectively. Curtain gas (Curtain Gas, CUR) was at 25 psi and the Collision-induced ionization (Collision-activated dissociation, CAD) parameter was set to high. QQQ scans were obtained as MRM experiments with collision gas (nitrogen) set to 5 psi. In the triple quadrupole (QQQ), DP and CE for individual MRM transitions was completed with DP and CE optimization. The resulting data was processed using the mass spectrometry software, Analyst (Version 1.6.1 Applied Biosystems Company, Framingham, MA, USA).

### 4.2. Qualitative and Quantitative Analysis of Metabolites

Qualitative analysis: Based on the MVDB V2.0 Database of Wuhan Maiteville Biotechnology Co., Ltd. (Wuhan, China), and the metabolite information public database, qualitative analysis of primary and secondary mass spectrometry data was obtained by referencing existing mass spectrometry databases such as MassBank, KNAPSAcK, HMDB, and METLIN; the structural analysis of metabolites was determined.

Quantitative analysis: Metabolites were quantified via the multiple reaction monitoring mode (MRM) using triple quadrupole mass spectrometry. In the MRM mode with detection window of 80 s and a target scan time of 1.5 s, the quadrupole filters the precursor ions (parent ions) of the target substance and excludes the ions corresponding to other molecular weights to prevent interference. After obtaining metabolite data from the different samples, the peak area of the mass spectra of all substances was integrated, and the mass spectra of the same metabolites in different samples were corrected.

### 4.3. Analysis of Metabolite Differences and Metabolic Pathways

To eliminate the effect of concentration differences on pattern recognition, the logarithm (log_10_) of the peak area matrix of black and white sesame seeds was obtained, and the Poisson normalization (subtraction of the mean square by the variance) was carried out. Principal component analysis (PCA), system clustering and data standardization, pattern recognition, and metabolic network analysis of the black sesame and white sesame metabolites were performed on the MetaboAnalyst 4.0 platform [41]. The analysis of metabolic pathways was achieved by using the KEGG metabolic pathway database, metabolite set enrichment analysis (MSEA) or pathway analysis (MetPA), and Pathway Topology Analysis.

## Figures and Tables

**Table 1:** Metabolic Pathway of black and white sesames. 217 out of 557 metabolites were putatively identified. Pathway analysis conducted at MetaboAnalyst 3.0 reflected that these 217 metabolites covered 30 pathways.

Metabolic Pathway	Total Number of Metabolites	Detected Metabolites
taurine and hypotaurine metabolism	5	1
alanine, aspartate and glutamate metabolism	22	5
arginine and proline metabolism	38	9
isoquinoline alkaloid biosynthesis	6	2
phenylalanine metabolism	8	2
tyrosine metabolism	18	2
tryptophan metabolism	27	7
sulfur metabolism	12	3
indole alkaloid biosynthesis	7	2
cysteine and methionine metabolism	34	8
glycine, serine and threonine metabolism	30	6
purine metabolism	61	11
riboflavin metabolism	10	1
phenylpropanoid biosynthesis	45	7
methane metabolism	11	1
pantothenate and CoA biosynthesis	14	2
flavonoid biosynthesis	43	2
aminoacyl-tRNA biosynthesis	67	19
pyrimidine metabolism	38	3
lysine degradation	17	2
glutathione metabolism	26	3
lysine biosynthesis	10	3
histidine metabolism	16	2
starch and sucrose metabolism	30	2
glycerophospholipid metabolism	25	3
galactose metabolism	26	1
valine, leucine and isoleucine biosynthesis	26	4
steroid biosynthesis	36	1
porphyrin and chlorophyll metabolism	29	2
selenoamino acid metabolism	19	2

**Table 2:** Significant differences of 20 metabolites in black and white sesame seeds. According to Volcano plot, if *p*-value is less than 0.01 and the fold change is greater than twice or less than half, significant difference of the metabolite exists between black sesame and white sesame.

Number	Compound	*p*	Number	Compound	*p*
1	indole-3-carboxylic acid	1.37 × 10^−8^	11	3-indoleacetonitrile	4.66 × 10^−5^
2	1-*O*-Sinapoyl-β-d-glucose	2.73 × 10^−7^	12	*N*-feruloylspermidine	5.28 × 10^−5^
3	hesperidin	3.50 × 10^−6^	13	tricetin 5-*O*-hexosyl-*O*-hexoside	8.43 × 10^−5^
4	2-methoxycinnamic acid	4.73 × 10^−6^	14	vanillin	1.8 × 10^−3^
5	thiamine	1.35 × 10^−5^	15	folic acid	1.9 × 10^−3^
6	vitamin B_2_	1.44 × 10^−5^	16	apigenin	2.4 × 10^−3^
7	coniferyl aldehyde	1.65 × 10^−5^	17	L-valine	3.2 × 10^−3^
8	ferulic acid	2.18 × 10^−5^	18	3-hydroxyanthranilic acid	4.2 × 10^−3^
9	phloretin	2.54 × 10^−5^	19	5-methoxy-*N*,*N*-dimethyltryptamine	7.0 × 10^−3^
10	hyoscyamine	2.59 × 10^−5^	20	l-cystathionine	9.6 × 10^−3^

## Supplementary Materials

The following are available online. Table S1: Identified metabolites of black and white sesames.
