# Supplementary material for: Identification of Nutritional Components in Black Sesame Determined by Widely Targeted Metabolomics and Traditional Chinese Medicines
Source: Molecules. 2018 May 15;23(5):1180. doi: 10.3390/molecules23051180 (PMC6100530; doi:10.3390/molecules23051180)
Supplement: Supplementary file 1 [file molecules-23-01180-s001.pdf]

Identification of Nutritional Components in Black Sesame Determined by Non-targeted Metabolomics and Traditional Chinese Medicines

Dandan Wang[1,2,§], Liangxiao Zhang[1,3,5,6,§*], Xiaorong Huang[1,2,§], Xiao Wang[1,5], Ruinan Yang[1,2], Jin Mao[1,5], Xuefang Wang[1,5], Xiupin Wang[1,5], Qi Zhang[1,4], Peiwu Li[1,3,4,5,*]

[1] Oil Crops Research Institute, Chinese Academy of Agricultural Sciences, Wuhan 430062, China

[2] Key Laboratory of Biology and Genetic Improvement of Oil Crops, Ministry of Agriculture, Wuhan 430062, China

[3] Laboratory of Quality and Safety Risk Assessment for Oilseed Products (Wuhan), Ministry of Agriculture, Wuhan 430062, China

[4] Key Laboratory of Detection for Mycotoxins, Ministry of Agriculture, Wuhan 430062, China

[5] Quality Inspection and Test Center for Oilseed Products, Ministry of Agriculture, Wuhan 430062, China

[6] Hubei Collaborative Innovation Center for Green Transformation of Bio-Resources, Wuhan 430062, China

[§] These authors contributed equally to this study

Supplementary materials:

Table S1 Identified metabolites of black and white sesames

Table 1 Identified metabolites of black and white sesames

Number	Metabolites	KEGG Entry
1	S-Adenosylhomocysteine	C00021
2	L-Glutamic acid	C00025
3	L-Alanine	C00041
4	L-Lysine	C00047
5	L-Aspartic acid	C00049
6	L-Arginine	C00062
7	L-Serine	C00065
8	L-Methionine	C00073
9	Ornithine	C00077
10	L-Tryptophan	C00078
11	L-Phenylalanine	C00079
12	L-Tyrosine	C00082
13	Sucrose	C00089
14	Glucose 6-phosphate	C00092
15	L-Cysteine	C00097
16	Choline	C00114
17	Biotin	C00120
18	L-Leucine	C00123
19	Inosinic acid	C00130
20	L-Histidine	C00135
21	Guanosine monophosphate	C00144
22	Adenine	C00147
23	L-Proline	C00148
24	L-Asparagine	C00152
25	Niacinamide	C00153
26	5'-Methylthioadenosine	C00170
27	Agmatine	C00179
28	L-Valine	C00183
29	Cholesterol	C00187
30	L-Threonine	C00188
31	Adenosine	C00212
32	10-Formyltetrahydrofolate	C00234
33	Guanine	C00242
34	Taurine	C00245
35	Nicotinic acid	C00253
36	Riboflavin (Vitamin B_2)	C00255
37	Inosine	C00294
38	Uridine	C00299

39	Pyridoxine	C00314
40	Spermidine	C00315
41	L-Carnitine	C00318
42	Glucosamine	C00329
43	Deoxyadenosine monophosphate	C00360
44	Cis-zeatin	C00371
45	Thiamine	C00378
46	Xanthine	C00385
47	Guanosine	C00387
48	Tryptamine	C00398
49	Tryptamine	C00398
50	L-Isoleucine	C00407
51	Pipecolic acid	C00408
52	5-Aminolevulinic acid	C00430
53	Saccharopine	C00449
54	Cytidine	C00475
55	Sinapic acid	C00482
56	Tyramine	C00483
57	Folic acid	C00504
58	Naringenin	C00509
59	Norepinephrine	C00547
60	Deoxyadenosine	C00559
61	p-Aminobenzoic acid	C00568
62	Phosphorylcholine	C00588
63	N-Acetylglutamic acid	C00624
64	2,5-dihydroxy benzoic acid	C00628
65	3-Hydroxyanthranilic acid	C00632
66	Oxitriptan	C00643
67	Mannitol 1-phosphate	C00644
68	Glycerophosphocholine	C00670
69	Betaine	C00719
70	Spermine	C00750
71	Vanillin	C00755
72	2,3-dihydroflavone	C00766
73	Phloretin	C00774
74	Serotonin	C00780
75	Creatinine	C00791
76	4-Hydroxycinnamic acid	C00811
77	4-Pyridoxic acid	C00847
78	L-Histidinol	C00860
79	Pantothenic acid	C00864
80	Sinapine	C00933
81	Aminoadipic acid	C00956
82	O-Acetylserine	C00979

83	Trigonelline	C01004
84	5-Hydroxy-L-tryptophan	C01017
85	Dimethylglycine	C01026
86	4-Guanidinobutanoic acid	C01035
87	Trehalose	C01083
88	Orotidylic acid	C01103
89	1-O-Sinapoyl-beta-D-glucose	C01175
90	Apigenin	C01477
91	Caffeic acid	C01481
92	trans-Ferulic acid	C01494
93	Linoleic acid	C01595
94	Kynurenic acid	C01717
95	Piperidine	C01746
96	Xanthosine	C01762
97	2-Hydroxycinnamic acid	C01772
98	Benzamidine	C01784
99	Homocystine	C01817
100	Methoxsalen	C01864
101	Homo-L-arginine	C01924
102	Acetylcholine	C01996
103	Hyoscyamine	C02046
104	L-Cystathionine	C02291
105	Phosphocreatine	C02305
106	5-Methylcytosine	C02376
107	Isonicotinamide	C02421
108	Xanthurenic acid	C02470
109	1-Methyladenosine	C02494
110	Coniferyl aldehyde	C02666
111	N-Acetylleucine	C02710
112	N-Acetylmuramic acid	C02713
113	Sinapoyl malate	C02887
114	1-Methylnicotinamide	C02918
115	3-Indoleacetonitrile	C02938
116	Argininosuccinic acid	C03406
117	trans-Zeatin-O-glucoside	C03423
118	2-Aminoisobutyric acid	C03665
119	Luteolin 7-glucoside	C03951
120	Chrysoeriol	C04293
121	5-(2-Hydroxyethyl)-4-methylthiazole	C04294
122	Protopine	C05189
123	Gamma-Glutamyl Glutamine	C05283
124	Ergothioneine	C05570
125	5-Hydroxyindoleacetic acid	C05635
126	5-Methoxytryptamine	C05659

127	5-Methoxyindoleacetate	C05660
128	Nicotinate D-ribonucleoside	C05841
129	Coumarin	C05851
130	Papaverine	C06533
131	Harmaline	C06536
132	Griseofulvin	C06686
133	Quinic acid	C06746
134	Amantadine	C06818
135	Gabapentin	C07018
136	Metaraminol	C07146
137	Feruloyl putrescine O-hexoside	C07166
138	Nandrolone	C07254
139	Pentamidine	C07420
140	Caffeine	C07481
141	Methoxamine	C07513
142	N-Acetylarylamine	C07565
143	5-Methoxydimethyltryptamine	C08309
144	5-Methoxy-N,N-dimethyltryptamine	C08309
145	Punicic acid	C08364
146	1H-Indole-3-carboxaldehyde	C08493
147	Cyanidin 3-glucoside	C08604
148	Diosgenin	C08898
149	Biflorin	C08996
150	Aesculin	C09264
151	Polygodial	C09712
152	Hesperidin	C09755
153	6-Prenylnaringenin	C09832
154	Tangeretin	C10190
155	Tricin	C10193
156	Eugenol	C10453
157	Gingerol	C10462
158	Subaphylline	C10497
159	7-Hydroxyflavone	C11264
160	4-methylumbelliferyl glucuronide	C11584
161	Chelidonine	C12242
162	Antipyrine	C13244
163	Xanthohumol	C16417
164	6-Methylmercaptopurine	C16614
165	Triethylenetetramine	C16700
166	Phenylglycine	C18623
167	N-Hydroxy-L-tryptophan	C19716
168	Indole-3-carboxylic acid	C19837
169	N-Acetylneuraminic acid	C19910
170	14,15-Dehydrocrepenynic acid	

171	2-Methoxycinnamic acid
172	3',4',5'-Tricetin 5-O-glucoside
173	4,6-Dihydroxyquinoline O-glucoside
174	4-Coumaroylcholine
175	4-Hydroxysphinganine
176	5,3'-Dihydroxyflavone
177	7-Benzyloxytryptamine
178	Caffeoyl shikimic acid
179	C-hexosyl-apigenin O-glucoside
180	C-hexosyl-luteolin O-glucoside
181	Chrysoeriol 5-O-glucoside
182	Chrysoeriol 7-O-glucoside
183	C-pentosyl-chrysoeriol O-glucoside
184	Dehydroabietylamine
185	Delphinidin 3-O-glucoside
186	Ferulic acid O-glucoside
187	Feruloyl quinate
188	Hesperetin 5-O-glucoside
189	Hesperidin methyl chalcone
190	Hydroxyphenethylamine
191	Kaempferol 3-O-glucoside
192	L-Methionine sulfone
193	Luteolin O-malonyl glucoside
194	Lycoperodine
195	Methyl Quercetin O-glucoside
196	N-Acetyl tryptamine
197	N-Benzoyl tryptamine
198	N-Feruloyl putrescine
199	N-Feruloyl agmatine
200	N-Feruloyl serotonin
201	N-Feruloyl spermidine
202	Nicotianamine
203	O-methyl Quercetin O-glucoside
204	p-Coumaroyl-2-hydroxyputrescine
205	Pelargonidin O-glucoside
206	Peonidin O-glucoside
207	Pyridoxine O-glucoside
208	Quercetin-3-beta-O-galactoside
209	Quercetin-3-O-rhamnoside
210	Tricetin 5-O-hexosyl-O-glucoside
211	Tricin 5-O-glucoside
212	Tricin 5-O-hexosyl-O-glucoside
213	Tricin 7-O-glucoside
214	Tricin O-hexosyl-O-glucoside

215	Tricin O-malonyl glucoside
216	Tuberonic acid glucoside
217	Xanthurenic acid O-glucoside
